# Electrochemical Heterogeneity at the Nanoscale: Diffusion to Partially Active Nanocubes

**DOI:** 10.1021/acs.jpclett.2c01922

**Published:** 2022-08-12

**Authors:** Rachel Wong, Christopher Batchelor-McAuley, Minjun Yang, Richard G. Compton

**Affiliations:** Physical and Theoretical Chemistry Laboratory Department of Chemistry, University of Oxford, Oxford OX1 3QZ, U.K.

## Abstract

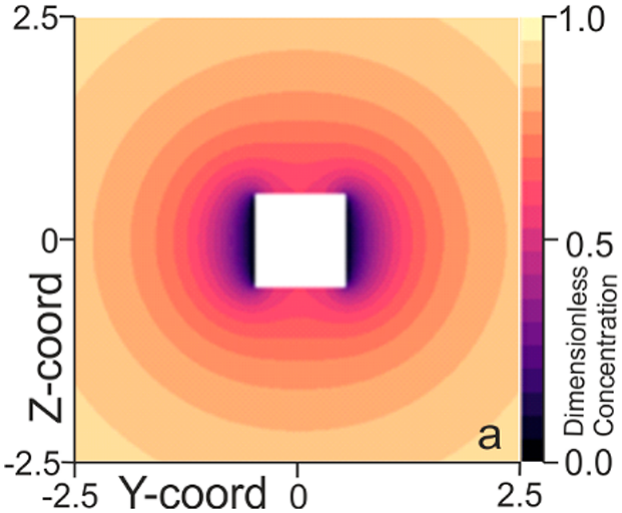

How does heterogeneity in activity affect the response of nanoparticles? This problem is key to studying the structure–activity relationship of new electrocatalytic materials. However, addressing this problem theoretically and to a high degree of accuracy requires the use of three-dimensional electrochemical simulations that have, until recently, been challenging to undertake. To start to probe this question, we investigate how the diffusion-limited flux to a cube changes as a function of the number of active faces. Importantly, it is clearly demonstrated how the flux is not linearly proportional to the active surface area of the material due to the faces of the cube not having diffusional independence, meaning that the flux to each face reflects the activity or not of nearby faces. These results have clear and important implications for experimental work that uses a correlation-based approach to evidence changes in activity at the nanoscale.

Electrochemical responses are generically intimately linked with mass transport, usually diffusion, prevailing in the vicinity of the electrode/solution interface of interest. This sensitivity is most obvious and simply apparent in the contrasting responses of macro- and microelectrodes where merely a change in the dimensions of an electrode made of the same material can alter a voltammetric response, such that greater overpotentials are needed to drive the reaction at smaller electrodes on account of the greater local rate of diffusion.^1−3^ More subtly, the response of a spatially heterogeneous electrode comprising of zones of different electrode activity depends not only on the absolute size of the different zones but also on the time scale of the experiment.^4^ Thus, at very short times they can respond almost independently of each other while at longer times the diffusional fields of the different parts can overlap with the consequence that the voltammetric response is dominated by the most active zones. In an extreme case this has the result that a relatively few active zones distributed over the surface of a macroelectrode can be sufficient for the voltammetry to reflect diffusion to the full geometric area of the electrode! This has very practical significance in the evaluation of nanomaterials where ultratrace impurities have been shown to give the illusion of electrocatalysis in diverse materials such as carbon nanotubes^5^ and graphene.^6^ Thus, traces of metals or metal oxides remaining from the synthesis of the nanomaterials have been shown to dominate the voltammetric signal leading to significant misinterpretation, for example, with respect to the activity of C_60_.^7^ Further and beyond the influence of trace impurities, presently a major focus of the electrochemical community is on development of experimental routes by which the structure/activity relationship of a material can be assessed.^8^ For some materials such as multilayered transition-metal dichalcogenides (TMDs)^9^ or other transition-metal structures such as MXenes,^10,11^ then just as the structure of the material is anisotropic so the chemical and catalytic properties of the “edge” or “basal” planes may also differ.^12,13^ Understanding and evidencing electrochemical heterogeneity are important across the field of electrocatalysis.

Conventional electrochemical techniques, such as voltammetry, measure the total current at a surface and consequently are unable, in this limit, to directly evidence heterogeneity in surface activity. This limitation has been the driving force for the development of techniques such as scanning electrochemical microscopy (SECM),^14^ scanning electrochemical cell microscopy (SECCM),^15^ and fluorescence electrochemical microscopy^16^ as routes by which heterogeneity in activity may be more readily experimentally probed. However, even though use of these techniques significantly improves the attainable spatial resolution to the submicrometer^17^ or even nanometer^14^ range, resolving heterogeneity in activity at the nanometer or even atomic level remains challenging. In the case of SECM and SECCM spatial resolution is limited by the size of the used probe, whereas for fluorescence microscopy diffusional blurring^18,19^ can be a significant issue. Hence, regardless of the used electrochemical technique, because of limitations in the spatial resolution, it is common that a *correlation-based* approach needs to be undertaken to yield greater insight into the physical origin of any observed catalytic effect. Underlying this is the assumption that if a nanostructure can be sufficiently well characterized, then through studying the catalytic reaction at the nanoscale it should at least in principle be possible to correlate the materials catalytic activity with its known structure and hence ascribe changed or enhanced activity to specific features of the material. The question is therefore to what extent is this true: if a nanoparticle exhibits heterogeneity in its catalytic activity, how successful can a correlation-based approach be in resolving the differing contributions to the overall flux if the spatial resolution is limited to the 100 nm or even 10 nm range? Even if we measure the activity of a single nanoparticle, can we directly relate the measured flux to its known structure? To start probing these questions, we herein consider the extreme case of a cubic particle in which only some of the particle’s surfaces are catalytically active and all of the other faces are perfectly inert. Such considerations are essential to guide experiments, now routinely made,^20^ electrochemically at the single (nano)particle or entity level by using the method of “nanoimpacts”.^21,22^ Note that the choice of a cube with faces of different activity permits the study of a complex particle with a level of numerical accuracy and precision appropriate for an electrochemical experiment (such as a nanoimpact experiment). The physical insight which emerges is that the faces are not diffusionally independent, and this will qualitatively apply to other nanoparticles such as tetrahedra and octahedra but which are geometries at the limit of present electrochemical simulations at the sought and required level of accuracy and precision. Nevertheless, the concept of diffusional nonindependence of the faces of any polyhedron of different levels of activity is general. Specifically, for example, if *m* of *n* faces is fully active, and (*n* – *m*) are inactive, then the total current flowing will be greater than *m*/*n* of the current seen if all faces are active. *This is for the simple reason that some of the electroactive material which would be electrolyzed on the nonactive faces if they were active can diffuse to one or other of the active faces as a result of the altered diffusion field*.

Here we consider diffusion to a cube and the mass-transport-limited flux to the entity when all or just some of the surface are active toward the reaction. Note that previous work^23^ has shown the steady-state flux (*J*/mol s^–1^) to an isolated cube of side length a (*m*) is if all faces of the cube are active:
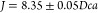
1Here *c* is the bulk concentration of the reactant (mol m^–3^), *D* is the molecular diffusion coefficient (m^2^ s^–1^), and *a* is the side length of the cube (m). This flux is the same as observed for a sphere of radius 1.34*a*/2, and it has been noted that the diffusion field around the cube becomes clearly spherical at surprisingly short distances away from the cube surface. We now pose the question of how the flux is altered if fewer than all size faces of the cube are “active”. Further we consider the idealized situation in which the particle is isolated in the solution phase and where the mass transport to/from the interface can be considered a diffusion-only process. By referring to the particle as “isolated”, we are stipulating that there is no other particle within its diffusion layer such that the flux to the interface is unperturbed. For an isolated nanoparticle the physically relevant diffusional case is that of the time-independent or steady-state response. At times greater than approximately *r*^2^/*D*, where *r* is the effective radius of the particle, then the diffusional profile is essentially constant near the interface. In the solution phase this means that for a submicro (nano)-sized particle the steady-state regime is reached after less than 1 ms. Consequently, in the following we focus on considering the steady-state flux to a particle with differing activity. We simulated the steady-state diffusional flux to a cubic particle by solving the time-independent diffusion equation using a 3D fully implicit finite difference method. More details on the simulation procedure can be found in the Computational Methods section. Because a fully implicit method is used, it is possible to directly calculate the steady-state flux without recourse to considering the time-dependent solution. At a semi-infinite distance from the cube surface the concentration of the reagent was set to its bulk value. At the nanoparticle surface two different boundary conditions were used: (a) the surface concentration of the reagent was set to zero, or (b) a zero flux boundary condition was used. These two boundaries represent the two extreme limits of (a) a perfectly catalytic surface or (b) a completely inert surface. Because of the simulation space being three-dimensional, solving the diffusion equation yields a three-dimensional concentration profile.

Herein, initially the flux to a cube with a single active face is considered. Figure 1a presents a single plane of the simulated three-dimensional concentration profile, and Figure 1b schematically indicates the plane being studied. The checkered plane shown in Figure 1b depicts the plane of the concentration profile shown in Figure 1a. Here we can see that at the active interface the concentration of the reagent is zero, and at distances further from the interface the concentration profile rapidly becomes more isotropic. Having obtained this concentration profile and through the use of Fick’s first law, it is possible to assess the total flux to the top face of the particle. Integrating across the entire catalytically active surface we find a steady-state flux of
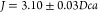
2where the terms in the expression are the same as those used previously in eq 1. It is instructive to first compare this rate to that expected for an isolated square surface imbedded in an infinite plane as has been previously reported.^24^ The flux found here for a cube with a single active face is ∼34% (3.1/2.31) greater than that to an equivalent square in a plane. This increase in the flux is not unreasonable as can be seen in Figure 1a; the catalytic surface consumes material from both above and below. Hence, for the single active surface of the cube there is more diffusionally accessible material as compared to the square surface in a plane leading to a higher flux density. In addition, it should be highlighted that although only one-sixth (i.e., ∼16%) of the cubic particles surface is active, the flux to this cube with a single active surface is ∼37% that of the entire active cube! In the case of the fully active cube the relatively lower flux density reflects the fact that the faces of the cube are not diffusionally independent; material consumed at one surface decreases the amount available at the adjacent interfaces.

**Figure 1:**
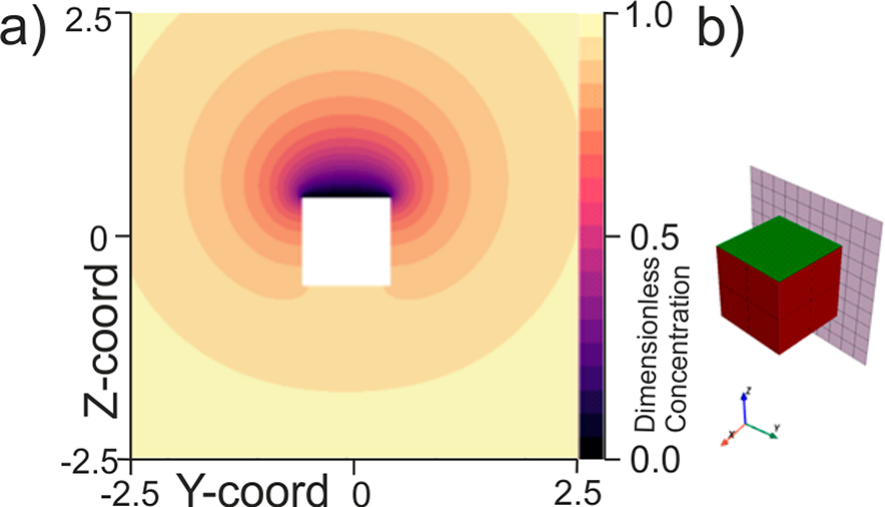
(a) Simulated concentration profile around a cubic particle at which only one of the faces (top face) is catalytically active. (b) Schematic indicating which plane of the concentration profile is being presented. The green face indicates an active surface, and the red face indicates an inactive face (no flux boundary).

Having considered this first simple case of one active surface, we now move to consider the steady-state concentration profile around a cubic particle in which two of its faces are active. We consider the case where the two active faces are nonadjacent. So as to more readily visualize the concentration profile here we again present a single plane of the concentration profile as shown in Figure 2a. Similarly, for clarity, the concentration profile that is being visualized in Figure 2a is schematically shown Figure 2b by the checkered plane that bisects the cube.

**Figure 2:**
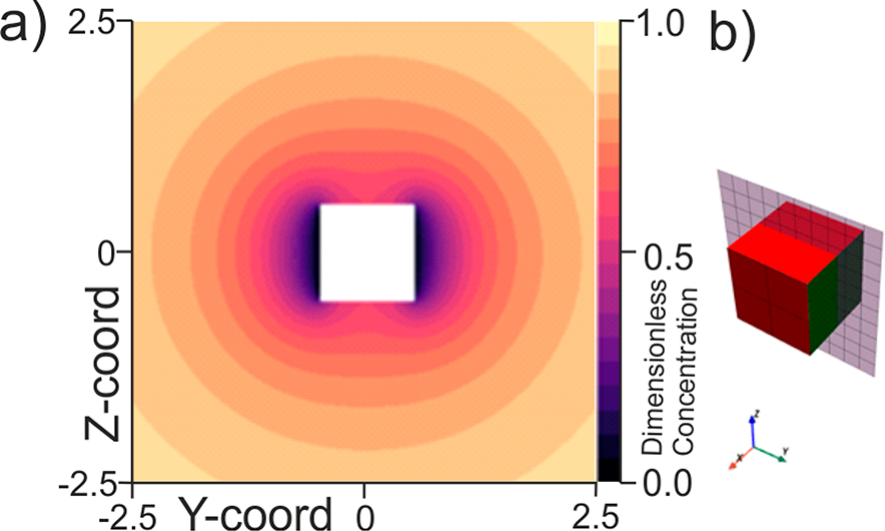
(a) Simulated concentration profile around a cubic particle at which only two of the faces (opposite faces) are catalytically active. (b) Schematic indicating which plane of the concentration profile is being depicted. The green face indicates an active surface, and the red face indicates an inactive face (no flux boundary).

Figure 2a shows how in the vicinity of the active surfaces the concentration of the solution phase species is significantly depleted. On moving away from the particle, the concentration profile rapidly becomes more isotropic. Here the total flux to the particle is found to be
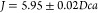
3This is an interesting result where through comparison of eq 3 with eq 1 we can see that although only one-third of the particle surface is active, the diffusion-limited flux is only ∼30% less than if the *entire* cube were catalytically active. Because of the fact that the flux to the cubic particle does not vary linearly as a function of the active surface area, this means that the flux density (mol m^–2^ s^–1^) varies as a function of both the particle size and the number of active surfaces. Perusal of Figure 2 shows that some of the material arriving at the active faces originates from a location close to an inactive face. This is the physical basis of the nonlinearity of the dependence of the current on the number of active faces because this is material that would be discharged on the inactive face from which it originated if the face were active. The easiest way to emphasize the degree to which the flux density is varying is to consider the average flux per active face. Note that across a single face the flux density is not uniform with there being a far higher proportion of the material consumed near the particle edges as has been previously discussed.^23^ For instance, in the present example where there are two active faces, the total dimensionless flux to the cubic particle is 5.95 ± 0.02 as stated in eq 3, but the average flux per face is 2.98 ± 0.01. This flux of 2.98 ± 0.01 is more than double that of the case in which the entire particle surface is active (1.39 ± 0.02).

Finally, to further explore and emphasize this nonlinear proportionality between the number of active surfaces and the total diffusion-limited flux to the cubic particle and the flux per face, a series of simulations were performed. Table 1 presents the simulated diffusion-limited flux to a cubic particle with a variable number of active surfaces. For situations in which more than one configuration of faces could be considered only the arrangement with this highest symmetry is considered. As can be seen from Table 1, as the number of active faces increases from one to six, the diffusion-limited flux density on the active surface decreases by a factor of 2.

**Table 1:** Simulated Dimensionless Flux and Dimensionless Flux Density (Presented as the Average Flux per Face) for a Partially Active Cubic Particle Where the Number of Active Faces Has Been Varied between 1 and 6^a^

no. of active faces on whole cube	total dimensionless flux	av flux per face
1	3.10 ± 0.03	3.10 ± 0.03
2	5.95 ± 0.02	2.98 ± 0.01
4	7.65 ± 0.03	1.91 ± 0.01
5	8.00 ± 0.02	1.60 ± 0.01
6	8.35 ± 0.05	1.39 ± 0.02

a In cases where more than one configuration of active faces exists we only consider the cubic particle with the highest symmetry.

As discussed, this change in the flux density is to be expected; the faces are not diffusionally independent, as increasing the active surface area decreases the relative availability of material and hence lowers the interfacial flux density. As can be seen in Figure 1, when material is consumed at a diffusion-limited rate at one face of the particle, a concentration gradient is formed, where the reagent is depleted over a distance that is comparable to the length scale of the face. In the case of one active face, material is even being consumed from the other side of the particle (as can be seen in the concentration profile which extends beyond the cube). As more active faces are added to the cube, the amount of material available for each new face is subsequently lower; this results in a decrease in the average flux per face and leads directly to the observed nonlinearity. The diffusional flux to the cubic particle is not linearly proportional to the active surface area. This nonlinearity is an important observation. For a nanoparticle with a heterogeneous surface, the particle and hence its activity are not under some, or potentially many, conditions—simply a “sum of its parts”—and the activity of the material needs to be considered in context. It should be further commented that due to the nonuniformity of the flux across the face of a cubic surface, even more extreme cases in which the only a small fraction of the particle being active may yield a total flux comparable to that of a fully active cube should be anticipated. For instance, in the situation where only the edges of the cube are active the total flux to the particle will likely be comparable to the flux to a fully active cube but only a relatively small fraction of the material may be active.

This work has considered the diffusion-limited flux to an isolated cubic particle where the faces of the particle are either fully active or completely inert. Importantly, it is demonstrated how the diffusion-limited flux to the material varies nonlinearly as a function of the number of active faces of the material. For instance, in the case where only one face of the cube is active (i.e., only ∼17% of the particle), then the total flux is 37% of the flux to a cube of the same size but where all of the surface is catalytically active. Hence, the average flux to this one face of the cube is over 2 times greater than the situation in which all faces of the cube are active. This result is extended to demonstrate how the total flux to the cube varies as a function of the number of active and inactive faces of the material. In the other limit where only one face of the cube is made inactive, the total flux to the cube is decreased by less than 5% even though ∼17% of the particle is now inactive. This nonlinearity in the flux as a function of active surface area is important and arises due to the faces of the cube not being diffusional independent; material consumed at one face decreases the amount of material available for the neighboring sites. This insight has potentially major implications for work in which researchers attempt to correlate the activity of a particle to its known or characterized structure. More succinctly, although correlation-based approaches to unraveling nanoparticle activity are one of the primary weapons in our arsenal to address fundamental questions relating to the origin of any given observed catalytic enhancement, it needs to be recognized that the flux and hence current to a surface is often not simply a linear combination of the active surfaces scaled relative to their surface area. The catalytic activity of a nanoparticle cannot in all—or possible many—cases be simply viewed as being a sum of its parts. This is potentially a major pitfall for any activity correlation-based methodology. Just as with the situation in which the activity of carbonaceous materials can be dominated by the presence of trace impurities, so too in the case with a nanoparticle a few highly active parts of the material may dominate the nanocatalytic response.

It is imperative that future work considers not only fluxes to heterogeneous particles of different geometries but also more importantly how heterogeneous particle activity will lead to changes in the measured particle activity far from the diffusion-limited region, in the so-called Tafel region, where surface concentrations of reactants are not significantly altered from that of the bulk solution. An open question remains: to what extent can we experimentally differentiate between a homogeneous particle of low overall activity versus a heterogeneous particle where only a small fraction of the surface is highly active? Ultimately the total particle flux as a function of potential is not unique for a given particle activity configuration; hence, care must be taken when using any correlation-based method to evidence change in particle activity. However, under diffusionally controlled electrolysis corresponding to highly electrocatalytic surfaces, the conclusions of this study are that the presence of some active faces can partly and significantly compensate for the presence of some inactive surfaces in the nanoparticle. The response is nonlinear in the number of active faces.

## Computational Methods

In this work we consider the steady-state diffusion-limited flux to a cubic particle isolated in the solution phase. We use a fully implicit finite difference methodology as developed in previous work^23,25^ and build on this theory to consider the situation in which not all of the cubic surface is active. Herein we succinctly outline the theory; further information regarding finite difference simulations can be found in the literature.^26^

We consider an irreversible interfacial reaction:
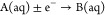
4where the reactant A is converted to species B via reduction or oxidation of A to B. To ensure the results in this work are general, we present the flux (*J*) in units of mol s^–1^. Conversion of this flux to an electrochemical current can be achieved by multiplying the flux by the number of electrons transferred (*n*) and the Faraday constant (96485 C mol^–1^). In addition, we will assume that the diffusion coefficients of species A and B are equal such that we only need to consider the concentration of one species as under these conditions and at all positions in space *c*_B_ = *c*_A_* – *c*_A_, where *c*_A_* is the initial concentration of species A.

As we will be considering a diffusion only problem, generally one needs to solve Fick’s second law (the diffusion equation) subject to appropriate boundary conditions. However, in this work we are concerned with the long time steady-state limit, in this case Fick’s second law reduces to the Laplace equation:
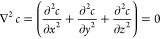
5where *c* is the concentration of the reagent and *x*, *y*, and *z* are the spatial coordinates. In this paper we study the flux of the reagent to a partially active cube. Because of the geometry of the cube, the concentration profile will be anisotropic, and as such the Laplace needs to be solved in all three spatial dimensions. Here we use Cartesian coordinates as indicated in eq 5. A simple expanding grid was used to discretize the simulation space. To solve this problem, the partial differential equation is subject to suitable boundary conditions. Initially, we will assume that only species A is in the solution and has a bulk concentration *c*_a_*. At a semi-infinite distance (set at 500 times the half-side length of the cube) from the cubes surface, the concentration of species A is also fixed to its bulk and initial value *c*_a_*. Most importantly, at the cube’s surface two differing boundary conditions are employed. If the surface is set as fully active, the surface concentration of species A is set as zero. Conversely, if the surface is set to be completely inert, a Neumann boundary condition is used and the flux at the surface is set to zero. In all simulation cases due to the symmetry of the problem, it was not necessary to consider the full cube. For example, when considering the flux to the fully active cube, only one octant of the space needs to be simulated; this is due to the planes of symmetry that are present such that the *xy*, *yz*, and *zx* planes in the simulation space are boundaries of zero flux. In addition, in the case of either one or five surfaces being active, then only a quarter of the simulation space needs to be considered as in this case only two of the three planes are a plane of symmetry. This reduction of the simulation space is beneficial in reducing the size of the numerical problem that needs to be solved. This is also the reason the activity of a particle with three active faces has been omitted; no reduction of the simulation space is possible in this case, leading to the full cube requiring simulation. This full cube 3D simulation is beyond the scope of the what is achievable with the present hardware as it requires more than 16 Gb of memory. Having solved the Laplace equation, the numerical simulation provides a full three-dimensional concentration profile in the vicinity of the particle. From this concentration profile the flux to the cube can be readily calculated; herein we use a simple two-point approximation to assess the magnitude of the interfacial flux.

This three-dimensional problem was discretized by using a central finite difference scheme and solved by using the HYPRE library,^27^ a highly optimized iterative sparse matrix solver. The program was written in C and compiled by using the NVCC compiler so as to allow it to be run on a GPGPU. The program was run on a Quadro GP100 graphics card (Nvidia, CA) which has 16 Gb of memory on a Linux machine with an Intel Core i7-6800 K CPU and 32 Gb of RAM. The required simulation times were less than 10 min for a fully converged solution. Convergence and benchmarking studies are reported in previous work.^23,25^

## Supporting Information Available

Transparent Peer Review report available (PDF)
