# Supplementary material for: Electrochemical Heterogeneity at the Nanoscale: Diffusion to Partially Active Nanocubes
Source: J Phys Chem Lett. 2022 Aug 12;13(33):7689–93. doi: 10.1021/acs.jpclett.2c01922 (PMC9421898; doi:10.1021/acs.jpclett.2c01922)
Supplement: Supplementary file 1 — jz2c01922_si_001.pdf [file jz2c01922_si_001.pdf]

jz-2022-01922k.R1

Name: Peer Review Information for "Electrochemical Heterogeneity at the Nanoscale: Diffusion to Partially Active Nanocubes"

First Round of Reviewer Comments

Reviewer: 1

Comments to the Author

This manuscript has investigated the relationship between the number of active surfaces in cubic electrocatalysts and the diffusion limited flux. The results demonstrated the diffusion limited flux is not linearly proportional to the active surface area of the material due to the faces of the cube not being diffusionally independent. The manuscript was well written and the discussions are in-depth and original, and could be potentially published in ***The Journal of Physical Chemistry Letters***. Nevertheless, several details remain to be clarified before I can recommend publication of this work, especially the difference and origin of catalytic activity.

1. In this work, the author considers the cube as a theoretical model. If it is a polyhedron with different morphologies, what will be the effect between the active surface and the related flux? The authors should consider more morphological nanoparticles such as tetrahedron, octahedron, dodecahedron and icosahedron, etc.

2. The authors present in Table 1 the simulated diffusion-limited fluxes of cubic particles with variable numbers of active surfaces. However, the number of 3 active faces on whole cube is not listed.

3. The authors mentioned that the diffusion limited flux to the material varies non-linearly as a function of the number of active faces of the material. However, the essential reason for this phenomenon is not given. More discussions about structure-activity relationship of new electrocatalytic materials should be added.

Reviewer: 2

Comments to the Author

This contribution reports a refreshing insight into the behavior of small particles in terms of the diffusion profiles that are established in the vicinity of their active parts. The authors address a very important issue with respect to the possible misinterpretation of measured currents as they show very convincingly that “less can be more”. In other words, they demonstrate that increasing the number of active sites (in the present model case represented by active planes of a cube) doesn’t necessarily lead to a linear increase in overall activity. For example, only a single plane can already lead to a flux that equals more than one-third of the total flux towards a fully active particle despite the fact that this surface only represents 1/6 of the total surface. This non-linear scaling is nicely illustrated in table 1. Even though these are only some rather preliminary results, I’m in favor of publication because the findings will certainly stimulate interesting discussions in the community of electrochemists, especially with respect to the characterization of electrocatalytic data. I would just ask the authors the avoid in the final version of the manuscript the slight redundancy which is currently present in the text when they discuss the possible wrong interpretation of activity in the presence of impurities etc… This is mentioned several times and should be streamlined. A tiny typo: in the caption of Figure 2 it should read “indicates an inactive face”.

Author's Response to Peer Review Comments:

DEPARTMENT OF CHEMISTRY

Physical and Theoretical Chemistry Laboratory
OX1 3QZ, UK
Tel: +44(0)1865 275957
Richard.Compton@chem.ox.ac.uk

***Reviewer #1:*** *In this work, the author considers the cube as a theoretical model. If it is a polyhedron with different morphologies, what will be the effect between the active surface and the related flux? The authors should consider more morphological nanoparticles such as tetrahedron, octahedron, dodecahedron and icosahedron, etc.*

This is a good point by the reviewer and studying the flux to other geometric shapes is presently being investigated by the authors. The full 3D simulation of the cube is numerically taxing and is close to the limit of what is achievable with the current hardware. Studying particles of lower symmetry requires more numerical processing and current work is focused on ensuring the convergence and accuracy of these numerical simulations. Consequently, at this time inclusion of other particle structures would be premature. A note has been added to the conclusion to highlight that future work should, and will, look to considering the behaviour of other geometries. That said, the use of other geometries will alter the specific details of the simulation but will not change the overall message- for heterogeneous nanoparticles the diffusive flux is not linearly proportional to the active surface area of the material.

- *The authors present in Table 1 the simulated diffusion-limited fluxes of cubic particles with variable numbers of active surfaces. However, the number of 3 active faces on whole cube is not listed.*

For a cube with three active surface there are two possible configurations of the faces. However, the symmetry of either configuration means that the entire particle needs to be simulated. None of the coordinate planes (XY,YZ or ZX) are planes of symmetry of these partially active cubes, such that the simulation space cannot be reduced in size. At present the simulation of a full 3D cube consumes more than the 16Gb of memory available on the graphics card. An explanation for the omission of the data has now been added to the computational methods section.

- *The authors mentioned that the diffusion limited flux to the material varies non-linearly as a function of the number of active faces of the material. However, the essential reason for this phenomenon is not given. More discussions about structure-activity relationship of new electrocatalytic materials should be added.*

Physically as mentioned by the reviewer in their summary of the paper (as part of the review), the reason for this non-linearity is due to the active faces not being diffusional independent. Further as explicitly clarified in the article this means that material consumed at one face decreases the amount of material available for the neighbouring faces, hence leading to the observed non-linearity. This aspect of the physical phenomenon is discussed and referenced at length in the introduction. However, to further emphasise the physical origin of this observation this following has been added after the discussion of Table 1.

> *"As can be seen in Figure 1 when material is consumed at a diffusion limited rate at one face of the particle a concentration gradient is formed, where the reagent is depleted over a distance that is comparable to the length scale of the face. In the case of one active face, material is even consumed from the other side of the particle (as can be seen in the concentration profile which extends beyond the cube). As more active faces are added to*

*the cube the amount of material available for each new face is subsequently lower, this results in a decrease in the average flux per face and leads directly to the observed non-linearity. The diffusional flux to the cubic particle is not linearly proportional to the active surface area."*

*Reviewer #2:*

We thank the referee for their very positive view of the work. We have now streamlined the conclusion of the paper such that we do not repeat the discussion regarding impurities. Further the typo in the caption of Figure 2 has been corrected.

jz-2022-01922k.R2

Name: Peer Review Information for "Electrochemical Heterogeneity at the Nanoscale: Diffusion to Partially Active Nanocubes"

Second Round of Reviewer Comments

Reviewer: 3

Comments to the Author

In this work, the authors investigated how the diffusion limited flux to a cube changes as a function of the number of active faces. They claimed they demonstrated how the flux is not linearly proportional to the active surface area of the material. I agree with Reviewer 1 that more morphological nanoparticles such as tetrahedron, octahedron, dodecahedron and icosahedron, should be considered here. This point is rather important to support their conclusions. However, I did not see any improvement on this point.

For the diffusion limited flux to the material variying non-linearly as a function of the number of active faces of the material, the underlying physics is not explained well.

In summary, the paper in the current form did not give new physical insight, I do not recommend its publicaiton.

Author's Response to Peer Review Comments:

Dear Editor,

Please find attached our response to the referee. We hope you find our manuscript is now suitable for publication at The Journal of Physical Chemistry Letters.

Yours sincerely,

Richard Compton

DEPARTMENT OF CHEMISTRY

Physical and Theoretical Chemistry Laboratory
OX1 3QZ, UK
Tel: +44(0)1865 275957
richard.compton@chem.ox.ac.uk

# Response to referee

Reviewer: 3

Recommendation: This paper is not recommended because it does not provide new physical insights.

Comments:
In this work, the authors investigated how the diffusion limited flux to a cube changes as a function of the number of active faces. They claimed they demonstrated how the flux is not linearly proportional to the active surface area of the material. I agree with Reviewer 1 that more morphological nanoparticles such as tetrahedron, octahedron, dodecahedron and icosahedron, should be considered here. This point is rather important to support their conclusions. However, I did not see any improvement on this point.

For the diffusion limited flux to the material variying non-linearly as a function of the number of active faces of the material, the underlying physics is not explained well.

In summary, the paper in the current form did not give new physical insight, I do not recommend its publicaiton.

Authors' response: The referee remains confused about the origin of diffusional non-independence. We have added the following to make this clearer along with amplifying the new physical insights.

> Page 2: "…having diffusional independence, meaning that the flux to each face reflects the activity or not of nearby faces."
>
> Page 4: "Note that the choice of a cube with faces of different activity permits the study of a complex particle with a level of numerical accuracy and precision appropriate for an electrochemical experiment (such as a nano-impact experiment). The physical insight which emerges is that the faces are not diffusionally independent and this will qualitatively apply to other nanoparticles such as tetrahedra, octahedra etc but which are geometries at the limit of present electrochemical simulations at the sought and required level of accuracy and precision. Nevertheless, the concept of diffusional non-independence of the faces of any polyhedron of different levels of activity is general. Specifically, for example, if m of n faces is fully active, and (n-m) are inactive then the total current flowing will be greater than m/n of the current seen if all faces are active. *This is for the simple reason that some of the electroactive material which would be electrolysed on the non- active faces if they were active is able to diffuse to one or other of the active faces as a result of the altered diffusion field.*"
>
> Page 7: "Perusal of Figure 2 shows that some of the material arriving at the active faces originates from a location close to an inactive face. This is the physical basis of the non-

DEPARTMENT OF CHEMISTRY

Physical and Theoretical Chemistry Laboratory
OX1 3QZ, UK
Tel: +44(0)1865 275957
richard.compton@chem.ox.ac.uk

> linearity of the dependence of the current on the number of active faces since this is material that would be discharged on the inactive face from which it originated if the face were active."
>
> Page 9: "However under diffusionally controlled electrolysis corresponding to highly electrocatalytic surfaces the conclusions of the present study are that the presence of some active faces can partly and significantly compensate for the presence of some inactive surfaces in the nanoparticle. The response is non-linear in the number of active faces."

She/he also suggested the study of other geometries. These are at the limit for the present simulations as explained on page 4 in the manuscript. But the key purpose of the present communication is the qualitative and quantitative new physical insight of diffusional non-independence of the faces of polyhedral nanoparticles, a result which does not need to await the evolution of improved simulations methods to allow more complex systems to be quantified.

We have made other minor corrections as highlighted and changed the TOC to outline the new physical insight reported.
